# Measurement Properties of Questionnaires Measuring Continuity of Care: A Systematic Review

**DOI:** 10.1371/journal.pone.0042256

**Published:** 2012-07-31

**Authors:** Annemarie A. Uijen, Claire W. Heinst, Francois G. Schellevis, Wil J.H.M. van den Bosch, Floris A. van de Laar, Caroline B. Terwee, Henk J. Schers

**Affiliations:** 1 Radboud University Nijmegen Medical Centre, Department of Primary and Community Care, Nijmegen, The Netherlands; 2 Netherlands Institute for Health Services Research (NIVEL), Utrecht, The Netherlands; 3 Department of General Practice and the EMGO Institute for Health and Care Research, VU University Medical Centre, Amsterdam, The Netherlands; 4 Department of Epidemiology and Biostatistics and the EMGO Institute for Health and Care Research, VU University Medical Centre, Amsterdam, The Netherlands; Bremen Institute of Preventive Research and Social Medicine, Germany

## Abstract

**Background:**

Continuity of care is widely acknowledged as a core value in family medicine. In this systematic review, we aimed to identify the instruments measuring continuity of care and to assess the quality of their measurement properties.

**Methods:**

We did a systematic review using the PubMed, Embase and PsycINFO databases, with an extensive search strategy including ‘continuity of care’, ‘coordination of care’, ‘integration of care’, ‘patient centered care’, ‘case management’ and its linguistic variations. We searched from 1995 to October 2011 and included articles describing the development and/or evaluation of the measurement properties of instruments measuring one or more dimensions of continuity of care (1) care from the same provider who knows and follows the patient (personal continuity), (2) communication and cooperation between care providers in one care setting (team continuity), and (3) communication and cooperation between care providers in different care settings (cross-boundary continuity). We assessed the methodological quality of the measurement properties of each instrument using the COSMIN checklist.

**Results:**

We included 24 articles describing the development and/or evaluation of 21 instruments. Ten instruments measured all three dimensions of continuity of care. Instruments were developed for different groups of patients or providers. For most instruments, three or four of the six measurement properties were assessed (mostly internal consistency, content validity, structural validity and construct validity). Six instruments scored positive on the quality of at least three of six measurement properties.

**Conclusions:**

Most included instruments have problems with either the number or quality of its assessed measurement properties or the ability to measure all three dimensions of continuity of care. Based on the results of this review, we recommend the use of one of the four most promising instruments, depending on the target population Diabetes Continuity of Care Questionnaire, Alberta Continuity of Services Scale-Mental Health, Heart Continuity of Care Questionnaire, and Nijmegen Continuity Questionnaire.

## Introduction

Continuity of care is an important characteristic of good health care. [1]–[4] In the literature, continuity often refers to the extent by which care is provided by the same person (personal continuity). Personal continuity is relatively easy to measure as it can be expressed as an index, based on duration of provider relationship, density of visits, dispersion of providers or sequence of providers [5].

From the 1990’s on, however, continuity of care is increasingly seen as a multidimensional concept. [6] Besides personal continuity, it also includes the seamless provision of care by a group of professionals in the medical home (team continuity), and continuity between different care settings, e.g. general practice and specialist care (cross-boundary continuity). [6]–[8] As more and more care providers are involved in individual patient care, the communication and cooperation aspects of care become increasingly important.

Measuring continuity of care in its multidimensional meaning requires a robust and solid measurement instrument. Reviews have shown that many instruments have been developed over time. [9]–[13] These reviews, however, did not include recent publications and have focused solely on one concept. As we found that other concepts like coordination and integration of care show great overlap with continuity of care [6], the limited continuity scope seems too narrow for a complete overview of instruments. Moreover, existing reviews have not systematically appraised the measurement properties of the instruments found. Therefore, we performed a systematic review to identify the instruments measuring continuity of care, to assess the dimensions of continuity in those instruments, and to evaluate their measurement properties.

## Methods

### Search Strategy

We searched the computerized bibliographic databases of PubMed, Embase and PsycINFO from 1995 to October 2011. We chose to start searching in 1995, as the multidimensional concept only emerged from then on. [6] It would therefore be very unlikely that relevant instruments developed before 1995 would use multidimensional definitions of continuity of care. We used the keywords ‘continuity of care’, ‘coordination of care’, ‘integration of care’, ‘patient centered care’, ‘case management’ and its linguistic variations in combination with a search filter developed for finding studies on measurement properties of measurement instruments (see Appendix S1). [14] We restricted our search to English or Dutch language articles. Reference lists were screened to identify additional relevant studies.

### Selection Criteria

We included all articles describing the development and/or evaluation of the measurement properties of an instrument measuring - what we will define in this review as - continuity of care [6]–[8]: (1) care from the same provider who knows and follows the patient (personal continuity), (2) communication and cooperation between care providers in one care setting (team continuity), and (3) communication and cooperation between care providers in different care settings (cross-boundary continuity). Instruments measuring only one or two of these dimensions were also included. Instruments based on a single item or index or instruments also measuring other concepts besides these three dimensions of continuity of care were excluded.

Two reviewers (AU and CH) independently screened titles, abstracts and reference lists of the studies retrieved by the literature search. If there was any doubt as to whether the article met the inclusion criteria, consensus was reached between the reviewers. The full-text articles were reviewed by two independent reviewers (AU and CH) for in- and exclusion criteria. If necessary a third independent reviewer (HS) was consulted.

### Data Extraction

Data extraction and assessment of measurement properties and methodological quality were performed by two reviewers (AU and CH) independently. In case of disagreement, a third reviewer (CT) made the decision. One of the found measurement instruments was developed and validated by AU [15]; [16], so CH and CT scored this instrument. All instruments were questionnaires with pre-defined answering categories. The following data were extracted:

1. Dimensions of continuity of care. For each questionnaire we identified which dimensions of continuity of care (personal, team and/or cross-boundary continuity) are measured.
2. Measurement properties. We describe the measurement properties of each questionnaire divided over three domains, according to the COSMIN taxonomy [17]: (1) reliability (including internal consistency, reliability, measurement error), (2) validity (including content validity, structural validity and hypothesis testing (construct validity)), and (3) responsiveness. These measurement properties are defined in Table 1. In addition, interpretability is also described. Interpretability is the degree to which one can assign qualitative meaning to quantitative scores. [17] This means that investigators should provide information about clinically meaningful differences in scores between subgroups, floor and ceiling effects, and the minimal important change. [18] Interpretability is not a measurement property, but an important characteristic of a measurement instrument [17].

**Table 1:** Quality criteria for measurement properties [23].

Property	Definition	Rating	Quality Criteria
**Reliability**	The degree to which scores for patients who have not changed are the same for repeated measurement under several conditions		
Internal consistency	The degree to which items in a (sub)scale are intercorrelated, thus measuring the same construct	+	+ (Sub)scale unidimensional AND Cronbach’s alpha(s) ≥0.70
		?	? Dimensionality not known OR Cronbach’s alpha not determined
		−	− (Sub)scale not unidimensional OR Cronbach’s alpha(s) <0.70
Reliability	The proportion of the total variance in the measurements which is because of ‘true’^a^ differences among patients	+	+ ICC/weighted Kappa ≥0.70 OR Pearson’s r≥0.80
		?	? Neither ICC/weighted Kappa, nor Pearson’s r determined
		−	− ICC/weighted Kappa <0.70 OR Pearson’s r<0.80
Measurement error	The systematic and random error of a patient’s score that is not attributed to true changes in the construct to be measured	+	+ MIC > SDC OR MIC outside the LOA
		?	? MIC not defined
		−	− MIC ≤ SDC OR MIC equals or inside LOA
**Validity**	The degree to which the instrument measures the construct(s) it purports to measure		
Content validity	The degree to which the content of an instrument is an adequate reflection of the construct to be measured	+	+ The target population considers all items in the questionnaire to be relevant AND considers the questionnaire to be complete
		?	? No target population involvement
		−	− The target population considers items in the questionnaire to be irrelevant OR considers the questionnaire to be incomplete
Structural validity	The degree to which the scores of an instrument are an adequate reflection of the dimensionality of the construct to be measured	+	+ Factors should explain at least 50% of the variance
		?	? Explained variance not mentioned
		−	− Factors explain <50% of the variance
Hypothesis testing(construct validity)	The degree to which the scores of an instrument are consistent with hypotheses (e.g. with regard to internal relationships, relationships to scores of other instruments, or differences between relevant groups) based on the assumption that the other instru	+	+ Correlation with an instrument measuring the same construct ≥0.50 OR at least 75% of the results are in accordance with the hypotheses AND correlation with related constructs is higher than with unrelated constructs
		?	? Solely correlations determined with unrelated constructs
		−	− Correlation with an instrument measuring the same construct <0.50 OR <75% of the results are in accordance with the hypotheses OR correlation with related constructs is lower than with unrelated constructs
**Responsiveness**			
Responsiveness	The ability of an instrument to detect change over time in the construct to be measured	+	+ (Correlation with an instrument measuring the same construct ≥0.50 OR at least 75% of the results are in accordance with the hypotheses OR AUC ≥0.70) AND correlation with related constructs is higher than with unrelated constructs
		?	? Solely correlations determined with unrelated constructs
		−	− Correlation with an instrument measuring the same construct <0.50 OR <75% of the results are in accordance with the hypotheses OR AUC <0.70 OR correlation with related constructs is lower than with unrelated constructs

a The word ‘true’ must be seen in the context of the classical test theory, which states that any observation is composed of two components - a true score and error associated with the observation. ‘True’ is the average score that would be obtained if the scale were given an infinite number of times. It refers only to the consistency of the score and not to its accuracy.

MIC  =  minimal important change, SDC  =  smallest detectable change, LOA  =  limits of agreement, ICC  =  intraclass correlation coefficient, AUC  =  area under the curve.

+  =  positive rating, ?  =  indeterminate rating, −  =  negative rating.

1. Quality assessment. Assessment of the methodological quality of the included studies was carried out using the COSMIN checklist. [19] This checklist consists of nine boxes with methodological standards for how each measurement property should be assessed. [20] Each item was rated on a 4-point scale (poor, fair, good or excellent). An overall score for the methodological quality of a study was determined by taking the lowest rating of any of the items in the nine boxes.

### Best Evidence Synthesis – Levels of Evidence

Some studies evaluated the same measurement properties for a specific questionnaire. To determine the overall quality of each measurement property established in different studies we combined the results of the different studies for each questionnaire, taking into account the number of studies, the methodological quality of the studies and the direction (positive or negative) and consistency of their results.

The possible overall rating for a measurement property could reach 8 different categories (+++, ++, +, +/−, ?, −, −− or −−−) [21]; [22] (Table 2). For example, when two studies of the same questionnaire show good methodological quality on evaluating ‘reliability’, then the overall rating would be either ‘+++’ or ‘−−−’ (Table 2), depending on the result (positive or negative) of the measurement property for which we used criteria based on Terwee et al. [23] (Table 1). These criteria were derived from existing guidelines and consensus within the research group of Terwee et al.

**Table 2:** Levels of evidence for the overall quality of the measurement property [22].

Rating	Criteria
+ + + or − − −	Consistent findings in multiple studies of good methodological quality OR in one study of excellent methodological quality
+ + or − −	Consistent findings in multiple studies of fair methodological quality OR in one study of good methodological quality
+ or −	One study of fair methodological quality
+/−	Conflicting findings
?	Only studies of poor methodological quality

+  =  positive rating, ?  =  indeterminate rating, −  =  negative rating.

In this case, when both studies showed intraclass correlation coefficient (ICC) <0.70, the overall rating would be ‘−−−’. This means that there is strong evidence (multiple studies of good methodological quality) for low levels of reliability. However, when there is only one study of fair methodological quality showing ICC>0.70, the overall rating would be ‘+’. When one study shows ICC>0.70, while another study shows ICC<0.70, the overall rating would be ‘+/−’. When there are only studies of poor methodological quality, the overall rating would be ‘?’, independent of the result of the measurement property.

## Results

**Figure 1:**
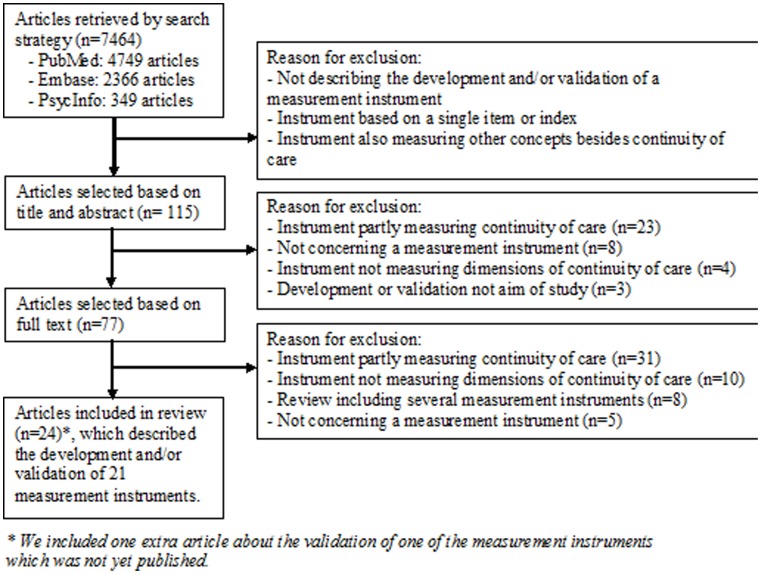
Search strategy resulting in 4749 articles from PubMed, 2366 articles from Embase and 349 articles from PsycInfo.

The search strategy resulted in 4749 articles from PubMed, 2366 articles from Embase and 349 articles from PsycInfo (Figure 1). From these searches, we included 23 articles in this review. We included one extra article that was not yet published which describes the validation of an included measurement instrument. [16] Reference tracking did not result in additional articles. Finally, we included 24 articles describing the development and/or evaluation of 21 questionnaires measuring continuity of care [15]; [16]; [24]–[45].

Table 3 presents an overview of the identified questionnaires. Seventeen questionnaires measured continuity of care from the perspective of the patien [15]; [16]; [24]–[27]; [29]–[35]; [37]–[41]; [43]–[45], four from the perspective of the care provider/program director [28]; [36]; [42]. From the instruments measuring continuity from the perspective of the patient, three were developed for diabetic patient [29]; [33]; [44], three for patients with a mental illnes [24]; [30]; [37]; [41]; [43], two for patients with cance [38]; [45], two for previously hospitalised patient [26]; [35], two for patients with complex and chronic care need [32]; [40], one for patients with heart failure or atrial fibrillatio [34]; [39], one for users of welfare services [25], one for patients visiting their family practice physician [31], one for patients living at home [27] and one for patients in general regardless of morbidity or care setting [15]; [16].

**Table 3:** Description of identified instruments.

Instrument	Reference number	Year of publication	Measurement aim	Target population	Language	No of items and subdomains	Response options	Domains of continuity of care
CPCI *(Components of Primary Care Index)*	31	1997	To measure several components of the delivery of primary care from the perspective of the patient	Patients visiting family practice physicians	English	19 items in 4 subdomains	5-point scale (range 1–5, mean factor scale score 1–5)	Personal continuity Team continuity Cross-boundary continuity
VCC *(Continuity of Care from client perspective)*	27	1998	To measure continuity of care from the patient perspective	Patients living at home	Dutch	126 items in 4 subdomains	5-point scale (range 1–5, total range 1–5)	Team continuity Cross-boundary continuity
CCI *(Care Continuity Instrument)*	26	2000	To measure continuity of care from the perspective of elders hospitalised for a chronic illness and their family caregivers	Elders hospitalised for a chronic illness	English	12 items in 4 subdomains	7-point scale (range 1–7, total range 12–84)	Personal continuity Team continuity Cross-boundary continuity
CONNECT	43	2003	To measure continuity of care for mental health services	Patients who have serious mental illness	English	59 items in 14 subdomains	5-point scale (range 1–5). Each subdomain was scored by summing the items and then rescaling to give a score out of 100	Team continuity Cross-boundary continuity
CPCQ *(Client Perceptions of Coordination Questionnaire)*	40	2003	To measure coordination of health care	Predominantly elderly patients with complex and chronic care needs	English	31 items in 7 subdomains	Most items were rated on a 5-point scale (range 1–5), 4 items were rated on a 3-point scale (range 1–3)	Team continuity Cross-boundary continuity
ACSS-MH *(Alberta Continuity of Services Scale – Mental Health)*	24; 30; 37	2004	To measure continuity of care for mental health services from the patient/client perspective	Patients using mental health services	English	32 items in 3 subdomains	5-point scale (range 1–5, mean factor scale score 1–5)	Personal continuity Team continuity Cross-boundary continuity
CCPS-I *(Continuity of Care Practices Survey – Individual level)*	42	2004	To measure the extent of continuity of care that staff (primary counselors/case managers) of substance use disorder programs provide to individual patients	Substance use disorder program staff (primary counselors/case managers)	English	23 items in 4 subdomains	Three subscales were scored on a 4-point scale, one subscale is scored as the mean of two percentages	Personal continuity Cross-boundary continuity
CCPS-P *(Continuity of Care Practices Survey – Program level)*	42	2004	To measure continuity of care from the perspective of substance use disorder program directors	Substance use disorder program directors	English	23 items in 4 subdomains	Three subscales were scored on a 4-point scale, one subscale is scored as the mean of two percentages	Personal continuity Cross-boundary continuity
DCCS *(Diabetes Continuity of Care Scale)*	29	2004	To measure continuity of care from the perspective of patients with diabetes	Diabetic patients	English	47 items in 5 subdomains	5-point scale (range 1–5, total score range 47–235)	Team continuity Cross-boundary continuity
HCCQ *(Heart Continuity of Care Questionnaire)*	34; 39	2004	To assess continuity of care from the perspective of patients with congestive heart failure and atrial fibrillation	Patients hospitalised for either congestive heart failure or atrial fibrillation	English	33 items in 3 subdomains	5-point scale (range 1–5, total range 1–5)	Personal continuity Team continuity Cross-boundary continuity
ECC-DM *(Experienced continuity of care for diabetes mellitus)*	33	2006	To measure continuity of care in type 2 diabetes mellitus	Type 2 diabetic patients	English	19 items in 4 subdomains	6-point scale. Each subdomain was scored by summing the items and then rescaling to give a score out of 25 (total score range 0–100).	Personal continuity Team continuity Cross-boundary continuity
King et al. (nameless instrument)	38	2008	To measure continuity of care in patients with cancer	Patients with cancer	English	18 items in 1 subdomain	5-point scale (range 0–4, total range 0–72)	Team continuity
CONTINU-UM *(Continuity of Care – User Measure)*	41	2008	To measure continuity of care in patients with severe mental illness	Patients who have severe mental illness	English	32 items in 16 subdomains	5-point scale (range unclear)	Personal continuity Team continuity Cross-boundary continuity
DCCQ *(Diabetes Continuity of Care Questionnaire)*	44	2008	To measure continuity of care in type 2 diabetes mellitus	Type 2 diabetic patients	Chinese	46 items in 8 subdomains	6-point scale, except for one subdomain (5-point scale). Each subdomain was scored by summing the items and then rescaling to give a score out of 100.	Personal continuity Team continuity Cross-boundary continuity
PCCQ *(Patient Continuity of Care Questionnaire)*	35	2008	To measure patient perceptions of factors impacting continuity of care following dischargefrom hospital	Patients previously hospitalised	English	27 items in 6 subdomains	5-point scale (range 1–5)	Personal continuity Team continuity Cross-boundary continuity
Ahgren et al. (nameless instrument)	25	2009	To assess the integration of welfare services from the perspective of the service users	Users of welfare services	Swedish	22 structured and open questions in 3 subdomains	The structured questions were rated on different ordinal scales (total range unclear)	Team continuity Cross-boundary continuity
CRP-PIM *(Communication with Referring Physicians Practice Improvement Module)*	36	2009	To assess the communication among physician consultants and referring physicians	Referring physicians	English	13 items in 2 subdomains	6-point scale (range 1–6)	Team continuity Cross-boundary continuity
CSI Survey *(Cancer Services Integration Survey)*	28	2009	To measure integration of cancer services	Healthcare providers and administrators that had regular opportunities to interact with the cancer system	English	54 items in 4 subdomains	5-point scale (range unclear)	Team continuity Cross-boundary continuity
Gulliford et al. (nameless instrument)	32	2011	To measure continuity of care from the perspective of patients with a long-term illness	Patients with a long-term ilness	English	16 items in 2 subdomains	4-point scale. In order to simpify further analysis, the authors used dichotomized item responses (0 or 1)	Personal continuity Team continuity Cross-boundary continuity
CCCQ *(Cancer Care Coordination Questionnaire)*	45	2011	To measure patients’ experience of cancer care coordination	Cancer patients in the treatment phase of the cancer journey	English	20 items in 2 subdomains	5-point scale (range 1–5, total range 20–100)	Team continuity Cross-boundary continuity
NCQ *(Nijmegen Continuity Questionnaire)*	15; 16	2011	To measure continuity of care from the patients’ perspective across primary and secondary care settings	All types of patients, regardless of care setting and morbidity	Dutch	28 items in 3 subdomains	5-point scale (range 1–5)	Personal continuity Team continuity Cross-boundary continuity

Ten instruments measured aspects of personal, team and cross-boundary continuit [15]; [16]; [24]; [26]; [30]–[35]; [37]; [39]; [41]; [44], while eleven instruments measured only one or two of these dimensions [25]; [27]–[29]; [36]; [38]; [40]; [42]; [43]; [45].

Most questionnaires were originally developed in English, except for the Dutch questionnaires of Casparie et al. [27] and Uijen et al. [15]; [16], the Chinese questionnaire of Wei et al. [44], and the Swedish questionnaire of Ahgren et al [25].

Table 4 presents a description of the study populations. Eight of the instruments were solely developed and/or evaluated in primary care population [27]; [31]–[33]; [40]; [41]; [43]; [44], eight solely in secondary care population [26]; [34]–[36]; [38]; [39]; [42]; [45] and five were developed and/or evaluated in both primary and secondary care populations [15]; [16]; [24]; [25]; [28]–[30]; [37].

**Table 4:** Description of identified study populations.

Article	Reference number	Instrument	Study population	Setting	N	Mean age (SD)	Male (%)	Country
Flocke	31	CPCI	Patients visiting family practice physicians	138 family practices	2899	42 (23)	38	USA
Casparie et al.	27	VCC	Patients living at home suffering from multiple sclerosis, rheumatoid artritis, astma, COPD, dementia or a mental impairment	Primary care	±1000		?	The Netherlands
Bull et al. (Phase I+ II)	26	CCI	Elders (>55 years) admitted to a community hospital for a chronic illness	Hospital	32	69.3 (8.9)	?	USA
Bull et al. (Phase III)	26	CCI	Elders (>55 years) recently hospitalized for an acute episode of congestive heart failure, chronic obstructive lung disease, or diabetes mellitus	Hospital	121	Range: 55–89 years	?	USA
Bull et al. (Phase IV)	26	CCI	Elders (>55 years) hospitalized with heart failure for at least two days	Hospital	135	74.1 (9.0)	?	USA
Ware et al.	43	CONNECT	Patients diagnosed with serious mental illness	Public mental health services	400	Range: 18–71 years	63	USA
McGuiness et al.	40	CPCQ	1. Patients with chronic complex health problems who could benefit from improved coordination of their health and social care 2. Patients with chronic pain	1. General practice 2. General practice and a community-based chronic pain management course	1380	59.1	39	Australia
Adair et al.	24	ACSS-MH	Patients in mental health services	Mental health services	317			Canada
Durbin et al.	30	ACSS-MH	Users of community and outpatient mental health programs	Mental health programs	215	25 years and younger: 6.6% 65+: 4.2%	37.9	Canada
Joyce et al.	37	ACSS-MH	Patients with a severe mental illness (psychotic disorder, bipolar disorder, or unipolar depressive disorder of at least 24 months duration)	Mental health services	441	42.5 (10.3)	41.0	Canada
Schaefer et al.	42	CCPS-I	Staff (primary counselors/case managers) of substance use disorder programs	Specialized mental health care	?	?	?	USA
Schaefer et al.	42	CCPS-P	Directors of different substance use disorder treatment programs	Specialized mental health care	117	?	?	USA
Dolovich et al.	29	DCCS	Patients with diabetes	A group health centre consisting of 33 family physicians and 31 specialists	60	60.8 (11.4)	56.7	Canada
Kowalyk et al.	39	HCCQ	Patients who had been hospitalized approximately six months earlier for either congestive heart failure or atrial fibrillation	Hospitals	83	74 (12)	56.6	Canada
Hadjistravropoulos et al.	34	HCCQ	Patients who had been hospitalized at least six months earlier for either congestive heart failure or atrial fibrillation	Hospitals	350	73.9 (range: 40–99 years)	54.0	Canada
Gulliford, Naithani et al.	33	ECC-DM	Patients with type 2 diabetes	19 family practices	193	65 (range: 32–90 years)	49.7	UK
King et al.	38	Nameless	Patients with breast, lung or colorectal cancer	National Cancer Networks	199	61.2 (11.8)	31.7	UK
Rose et al.	41	CONTINU-UM	Patients who had a diagnosis of psychosis and had been in touch with services for at least 2 years	Community mental health teams	167	43	56	UK
Wei et al.	44	DCCQ	Patients with type 2 diabetes	Community health centre	338	68.7 (9.7)	32.2	China
Hadjistravropoulos et al.	35	PCCQ	Patients discharged from either an orthopaedics unit or a family medicine unit	Hospitals	204	64.9 (17.4)	40.2	Canada
Ahgren et al.	25	Nameless	Users of different institutions in the rehabilitation field that provide services to people who have been ill or unemployed for a long time	Institutions in the rehabilitation field	454	40	40	Sweden
Hess et al.	36	CRP-PIM	Physicians referring to consultants (internists and subspecialists)	Hospital	12212	47 (3.9)	76	USA
Dobrow et al.	28	CSI	Healthcare providers and administrators that had regular opportunities to interact with the cancer system	Hospitals and community care access centres	1769	Between 40–60: 71%	31.0	Canada
Gulliford, Cowie et al.	32	Nameless	Patients aged 60 years or older	General practice	1125	?	45.5	UK
Young et al.	45	CCCQ	1. Patients in follow-up for any cancer that had been treated 3–12 months previously 2. Patients with a newly diagnosed colorectal cancer	Hospital	686	66.1 (13.3)	53.2	Australia
Uijen, Schellevis et al.	15	NCQ	Patients with one or more chronic diseases	General practice	288	64.6	46.2	The Netherlands
Uijen, Schers et al.	16	NCQ	Patients with one or more chronic diseases	General practice and hospital/outpatient department	268	62.2	48.5	The Netherlands

The methodological quality of the studies is presented in Table 5 for each questionnaire and measurement property. Most studies assessed the internal consistency, content validity, structural validity and construct validity of the instruments, although frequently the methodological quality of the studies regarding these measurement properties was fair or poor. The reliability and measurement error were only assessed in a minority of the studies and the methodological quality regarding these measurement properties was often fair or poor. Cross-cultural validity, criterion validity and responsiveness were not assessed in any of the studies.

**Table 5:** Methodological quality of each article per measurement property and instrument (COSMIN Checklist).

Article	Reference number	Internal Consistency	Reliability	Measurement Error	Content Validity	Structural Validity	Hypotheses Testing
CPCI							
Flocke	31	Good	−	−	Excellent	Good	Fair
VCC							
Casparie et al.	27	Good	−	−	Excellent	Good	−
CCI							
Bull et al. (Phase I+II)	26	Poor	−	−	Fair	−	Fair
Bull et al. (Phase III)	26	Excellent	−	−	−	Excellent	Good
Bull et al. (Phase IV)	26	Excellent	Excellent	−	−	Excellent	Fair
CONNECT							
Ware et al.	43	Poor	Good	−	Good	−	Poor
CPCQ							
McGuiness et al.	40	Excellent	−	−	Fair	Fair	Fair
ACSS-MH							
Adair et al.	24	Fair	Fair	−	Excellent	Fair	−
Durbin et al.	30	Excellent	−	−	−	Excellent	Fair
Joyce et al.	37	Good	−	−	−	Good	Fair
CCPS-I							
Schaefer et al.	42	Poor	−	−	Poor	−	−
CCPS-P							
Schaefer et al.	42	Poor	−	−	Fair	−	Poor
DCCS							
Dolovich et al.	29	Poor	Fair	−	Fair	Poor	Fair
HCCQ							
Kowalyk et al.	39	Poor	−	−	Fair	−	Good
Hadjistravropoulos et al.	34	Excellent	−	−	−	Good	Good
ECC-DM							
Gulliford, Naithani et al.	33	Excellent	−	Poor	−	Good	Poor
King et al. (Nameless)							
King et al.	38	Poor	Fair	−	Excellent	−	−
CONTINU-UM							
Rose et al.	41	−	Fair	Fair	Poor	−	−
DCCQ							
Wei et al.	44	Fair	−	−	Fair	Poor	Fair
PCCQ							
Hadjistravropoulos et al.	35	Poor	−	−	Poor	Poor	Good
Ahgren et al. (Nameless)							
Ahgren et al.	25	Poor	−	−	Fair	−	−
CRP-PIM							
Hess et al.	36	−	Poor	−	−	Fair	−
CSI							
Dobrow	28	Poor	−	−	Excellent	Poor	−
Gulliford et al. (nameless)							
Gulliford, Cowie et al.	32	Fair	−	−	Poor	Fair	−
CCCQ							
Young et al.	45	Excellent	Excellent	−	Excellent	Excellent	Poor
NCQ							
Uijen, Schellevis et al.	15	Excellent	−	−	Fair	Poor	−
Uijen, Schers et al.	16	Excellent	Excellent	Excellent	−	Poor	Excellent

Cross-cultural validity, criterion validity and responsiveness were not evaluated

−: no information available.

The synthesis of results per questionnaire and their accompanying level of evidence are presented in Table 6. Six instruments (CPCI [31], CCI [26], CPCQ [40], HCC [34]; [39], CCCQ [45] and NC [15]; [16]) scored positive on the quality of at least three measurement properties. Information regarding the interpretability of the instruments was missing in most studies.

**Table 6:** Quality of measurement properties and the interpretability per instrument.

	Measurement properties						Interpretability			
Instrument	Internal Consistency	Reliability	Measurement Error	Content Validity	Structural Validity	Hypotheses Testing	Differences in scores between subgroups	Floor/ceiling effects of subdomain(s)	Minimal important change (MIC)	
CPCI	− −	na	na	+ + +	+ +	+	Not reported	Unknown	Unknown	4, 3 positief
VCC	− −	na	na	+ + +	+ +	na	Not reported	Unknown	Unknown	3, 2 positief
CCI	+ + +	− − −	na	+	+ + +	+/−	Not reported	Floor and ceiling effect	Unknown	5, 3 positief
CONNECT	?	− −	na	+ +	na	?	Not reported	Floor effect	Unknown	4, 1 positief
CPCQ	− − −	na	na	+	+	+	Not reported	Unknown	Unknown	4, 3 positief
ACSS-MH	+/−	−	na	+ + +	− − −	+	Reported	Unknown	Unknown	5, 2 positief
CCPS-I	?	na	na	?	na	na	Not reported	Unknown	Unknown	2, 0 positief
CCPS-P	?	na	na	+	na	?	Not reported	Unknown	Unknown	3, 1 positief
DCCS	?	+	na	+	?	−	Reported	Ceiling effect	Unknown	5, 2 positief
HCCQ	+ + +	na	na	+	− −	+ + +	Not reported	Unknown	Unknown	4, 3 positief
ECC-DM	− − −	na	?	na	+ +	?	Reported	Unknown	Unknown	4, 1 positief
King et al. (Nameless)	?	+	na	+ + +	na	na	Not reported	Unknown	Unknown	3, 2 positief
CONTINU-UM	na	+	?	?	na	na	Not reported	Unknown	Unknown	3, 1 positief
DCCQ	+	na	na	+	?	−	Not reported	No floor/ceiling effect	Unknown	4, 2 positief
PCCQ	?	na	na	?	?	+ +	Reported	Unknown	Unknown	4, 1 positief
Ahgren et al. (Nameless)	?	na	na	+	na	na	Not reported	Unknown	Unknown	2, 1 positief
CRP-PIM	na	?	na	na	−	na	Not reported	Ceiling effect	Unknown	2, 0 positief
CSI	?	na	na	+ + +	?	na	Not reported	No floor/ceiling effect	Unknown	3, 1 positief
Gulliford et al. (nameless)	+	na	na	?	+	na	Reported	Unknown	Unknown	3, 2 positief
CCCQ	+ + +	− − −	na	+ + +	+ + +	?	Not reported	Ceiling effect	Unknown	5, 3 positief
NCQ	+ + +	+ + +	?	+	?	+ + +	Reported	No floor/ceiling effect	Unknown	6, 4 positief

+++ or −−−  =  strong evidence positive/negative result, ++ or −  =  moderate evidence positive/negative result, + or −  =  limited evidence positive/negative result, +/−  =  conflicting evidence, ?  =  unknown, due to poor methodological quality.

na  =  no information available.

Cross-cultural validity, criterion validity and responsiveness were not evaluated.

## Discussion

In this systematic review we found 21 instruments measuring - what we define as - continuity of care. We found six instruments that we would probably not have found when we would have focussed our review solely on continuity of care, instead of taking into account related concepts as coordination and integration. [25]; [28]; [31]; [36]; [40]; [45] CPCQ and CCCQ aim to measure ‘coordination of care’ [40]; [45], CSI and the instrument of Ahgren et al. measure ‘integration of care’ [25]; [28], CRP-PIM measures ‘communication among care providers’ [36] and CPCI measures ‘attributes of primary care’ [31].

Most included instruments have problems with either the ability to measure all three dimensions of continuity of care or the number or quality of its assessed measurement properties.

Only about half of the questionnaires measured all three dimensions of continuity of care (personal, team and cross-boundary continuity). Of most instruments three or four measurement properties were assessed (mostly internal consistency, content validity, structural validity and construct validity). Only six instruments (CPCI [31], CCI [26], CPCQ [40], HCCQ [34]; [39], CCCQ [45] and NCQ [15]; [16]) scored positive on the quality of at least three measurement properties. These findings do not mean that the other questionnaires are of poor quality, but imply that studies of high methodological quality are needed to properly assess their measurement properties.

### Strengths and Limitations

One of the strengths of this review is that our search not only focused on the concept of ‘continuity of care’, but also took into account the relating concepts ‘coordination of care’, ‘integration of care’, ‘case management’ and ‘patient centred care’. This resulted in the inclusion of instruments which measure the same aspects of care but are defined in different ways.

To our knowledge, this is the first review on measurement instruments for continuity of care that systematically appraised the measurement properties of the instruments found. This allows us to compare the instruments on the quality of their measurement properties.

We used a robust and standardized method to assess the quality of the measurement properties, which attributes considerably to the continuity knowledge base.

A limitation of this study is that we searched from 1995 onwards. Measurement instruments developed before this time were not included in our review. However, because of the changing definitions of continuity over time, we consider it very unlikely that we missed relevant instruments [6].

Another limitation is that the raters had to make a large number of judgements on each study and each measurement instrument. Although the COSMIN checklist [19] and the quality criteria for the measurement properties [23] are defined as objective as possible, different raters could come to a different judgement. That is why two reviewers assessed the measurement properties and methodological quality of the studies, and in case of disagreement a third reviewer was consulted.

### Comparison with Existing Literature

Previous reviews have identified many instruments measuring continuity of care or one of its related concepts, such as patient centred care or integrated care. [9]–[13] Most reviews have limited their search to only one concept. We found only one review, identifying measures of integrated care, that broadened its search to concepts as continuity of care, care coordination and seamless care, but this review did not systematically appraise quality measures of the instruments. [13] Most instruments included in previous reviews have not been included in our review due to several reasons. Some studies did not describe the development or evaluation of the measurement properties at all, some did not measure - what we define in this review as - continuity of care, and some measured a much broader concept than continuity of care (e.g. all key areas of primary care including accessibility and thoroughness of physical examination).

We found no review assessing the quality of the measurement properties of the included instruments. Hudon et al. systematically assessed the quality of the included articles, i.e. whether all relevant information such as characteristics of the study population was described. [10] However, the quality of the measurement properties was not assessed.

### Implications for Practice and Research

The decision which instrument to use will depend on the characteristics of the study population, the ability and desire to measure all three dimensions of continuity, the population in which the instrument was developed and/or validated, the quality of the measurement properties and the interpretability of the instrument.

For a comprehensive measurement of continuity of care, we recommend to use the the DCCQ [44] for diabetic patients, as both other questionnaires for diabetic patients (DCCS [29] and ECC-DM [33]) either do not measure all three dimensions of continuity of care or show lower quality of their measurement properties and interpretability.

For patients with a mental illness, we recommend to use the the ACSS-MH [24]; [30]; [37]. Both other questionnaires available for patients with a mental illness (CONNECT [43] and CONTINU-UM [41]) are only validated in primary care, do not measure all three dimensions of continuity of care or show lower quality of their measurement properties and interpretability.

For patients with heart failure or atrial fibrillation, we only found the HCC [34]; [39]. As this instrument measures relational, team and cross-boundary continuity and shows good quality of the measurement properties, this seems to be a proper questionnaire for this patient group.

For patients with a (chronic) illness (irrespective of the type of (chronic) illness), we found the CPCI [31], VCC [27], CPCQ [40], the instrument of Gulliford et al. [32] and the NCQ [15]; [16]. For a comprehensive measurement of continuity of care, the NCQ is the only questionnaire that has been validated in primary and secondary care and shows the highest quality of its measurement properties and interpretability.

The instruments developed to measure continuity for patients with cancer (CCCQ [45] and the instrument of King et al. [38]), patients previously hospitalized (CCI [26] and PCCQ [35]), and users of welfare services (instrument of Ahgren et al. [25]) all have problems regarding the limited number of dimensions of continuity measured, the limited quality of the measurement properties or the low interpretability of the instrument. The instruments developed to measure continuity of care from the perspective of the provider (CCPS-I [42], CCPS-P [42], CRP-PIM [36] and CSI [28]) need to be used with caution because of the limited quality of the measurement properties and interpretability.

For future research, we believe it is especially important to further evaluate the measurement properties and interpretability of the promising DCCQ, ACSS-MH, HCCQ and NCQ. For none of these instruments, responsiveness is evaluated, although this is an important characteristic of a questionnaire, especially when used to measure change in continuity of care. As the DCCQ and NCQ are originally developed in respectively Chinese and Dutch, cross-cultural validation needs to be evaluated.

## Supporting Information

Appendix S1 **Search strategy.**
(DOCX)
