# Supplementary material for: Measurement Properties of Questionnaires Measuring Continuity of Care: A Systematic Review
Source: PLoS One. 2012 Jul 31;7(7):e42256. doi: 10.1371/journal.pone.0042256 (PMC3409169; doi:10.1371/journal.pone.0042256)
Supplement: Appendix S1 — Search strategy. (DOCX) [file pone.0042256.s001.docx]

**Appendix**

**Search strategy Pubmed**

“Continuity of care”[tiab] OR “Continuity of health care”[tiab] OR “Continuity of healthcare”[tiab] OR “Continuity of patient care”[tiab] OR “Continuity of service”[tiab] OR “Continuity of services”[tiab] OR “Care continuity”[tiab] OR “Healthcare continuity”[tiab] OR “Service continuity”[tiab] OR “Services continuity”[tiab] OR “Coordination of care”[tiab] OR “Coordination of health care”[tiab] OR “Coordination of healthcare”[tiab] OR “Coordination of patient care”[tiab] OR “Coordination of service”[tiab] OR “Coordination of services”[tiab] OR “Care coordination”[tiab] OR “Healthcare coordination”[tiab] OR “Service coordination”[tiab] OR “Services coordination”[tiab] OR “Co-ordination of care”[tiab] OR “Co-ordination of health care”[tiab] OR “Co-ordination of healthcare”[tiab] OR “Co-ordination of patient care”[tiab] OR “Co-ordination of service”[tiab] OR “Co-ordination of services”[tiab] OR “Care co-ordination”[tiab] OR “Healthcare co-ordination”[tiab] OR “Service co-ordination”[tiab] OR “Services co-ordination”[tiab] OR “Coordinated care”[tiab] OR “Coordinated health care”[tiab] OR “Coordinated healthcare”[tiab] OR “Coordinated patient care”[tiab] OR “Coordinated service”[tiab] OR “Coordinated services”[tiab] OR “Co-ordinated care”[tiab] OR “Co-ordinated health care”[tiab] OR “Co-ordinated healthcare”[tiab] OR “Co-ordinated patient care”[tiab] OR “Co-ordinated service”[tiab] OR “Co-ordinated services”[tiab] OR “Coordinating care”[tiab] OR “Coordinating health care”[tiab] OR “Coordinating healthcare”[tiab] OR “Coordinating patient care”[tiab] OR “Coordinating service”[tiab] OR “Coordinating services”[tiab] OR “Co-ordinating care”[tiab] OR “Co-ordinating health care”[tiab] OR “Co-ordinating healthcare”[tiab] OR “Co-ordinating patient care”[tiab] OR “Co-ordinating service”[tiab] OR “Co-ordinating services”[tiab] OR “Integration of care”[tiab] OR “Integration of health care”[tiab] OR “Integration of healthcare”[tiab] OR “Integration of patient care”[tiab] OR “Integration of service”[tiab] OR “Integration of services”[tiab] OR “Care integration”[tiab] OR “Healthcare integration”[tiab] OR “Service integration”[tiab] OR “Services integration”[tiab] OR “Integrated care”[tiab] OR “Integrated health care”[tiab] OR “Integrated healthcare”[tiab] OR “Integrated patient care”[tiab] OR “Integrated service”[tiab] OR “Integrated services”[tiab] OR “Integrating care”[tiab] OR “Integrating health care”[tiab] OR “Integrating healthcare”[tiab] OR “Integrating patient care”[tiab] OR “Integrating service”[tiab] OR “Integrating services”[tiab] OR “Patient centered care”[tiab] OR “Patient centered health care”[tiab] OR “Patient centered healthcare”[tiab] OR “Patient centered service”[tiab] OR “Patient centered services”[tiab] OR “Patient centred care”[tiab] OR “Patient centred health care”[tiab] OR “Patient centred healthcare”[tiab] OR “Patient centred service”[tiab] OR “Patient centred services”[tiab] OR “Patient focused care”[tiab] OR “Patient focused health care”[tiab] OR “Patient focused healthcare”[tiab] OR “Patient focused service”[tiab] OR “Patient focused services”[tiab] OR “Case management”[tiab]

AND

(instrumentation[sh] OR methods[sh] OR Validation Studies[pt] OR Comparative Study[pt] OR “psychometrics”[MeSH] OR psychometr*[tiab] OR clinimetr*[tw] OR clinometr*[tw] OR “outcome assessment (health care)”[MeSH] OR outcome assessment[tiab] OR outcome measure*[tw] OR “observer variation”[MeSH] OR observer variation[tiab] OR “Health Status Indicators”[Mesh] OR “reproducibility of results”[MeSH] OR reproducib*[tiab] OR “discriminant analysis”[MeSH] OR reliab*[tiab] OR unreliab*[tiab] OR valid*[tiab] OR coefficient[tiab] OR homogeneity[tiab] OR homogeneous[tiab] OR “internal consistency”[tiab] OR (cronbach*[tiab] AND (alpha[tiab] OR alphas[tiab])) OR (item[tiab] AND (correlation*[tiab] OR selection*[tiab] OR reduction*[tiab])) OR agreement[tiab] OR precision[tiab] OR imprecision[tiab] OR “precise values”[tiab] OR test–retest[tiab] OR (test[tiab] AND retest[tiab]) OR (reliab*[tiab] AND (test[tiab] OR retest[tiab])) OR stability[tiab] OR interrater[tiab] OR inter-rater[tiab] OR intrarater[tiab] OR intra-rater[tiab] OR intertester[tiab] OR inter-tester[tiab] OR intratester[tiab] OR intra-tester[tiab] OR interobserver[tiab] OR inter-observer[tiab] OR intraobserver[tiab] OR intra-observer[tiab] OR intertechnician[tiab] OR inter-technician[tiab] OR intratechnician[tiab] OR intra-technician[tiab] OR interexaminer[tiab] OR inter-examiner[tiab] OR intraexaminer[tiab] OR intra-examiner[tiab] OR interassay[tiab] OR inter-assay[tiab] OR intraassay[tiab] OR intra-assay[tiab] OR interindividual[tiab] OR inter-individual[tiab] OR intraindividual[tiab] OR intra-individual[tiab] OR interparticipant[tiab] OR inter-participant[tiab] OR intraparticipant[tiab] OR intra-participant[tiab] OR kappa[tiab] OR kappa’s[tiab] OR kappas[tiab] OR repeatab*[tiab] OR ((replicab*[tiab] OR repeated[tiab]) AND (measure[tiab] OR measures[tiab] OR findings[tiab] OR result[tiab] OR results[tiab] OR test[tiab] OR tests[tiab])) OR generaliza*[tiab] OR generalisa*[tiab] OR concordance[tiab] OR (intraclass[tiab] AND correlation*[tiab]) OR discriminative[tiab] OR “known group”[tiab] OR factor analysis[tiab] OR factor analyses[tiab] OR dimension*[tiab] OR subscale*[tiab] OR (multitrait[tiab] AND scaling[tiab] AND (analysis[tiab] OR analyses[tiab])) OR item discriminant[tiab] OR interscale correlation*[tiab] OR error[tiab] OR errors[tiab] OR “individual variability”[tiab] OR (variability[tiab] AND (analysis[tiab] OR values[tiab])) OR (uncertainty[tiab] AND (measurement[tiab] OR measuring[tiab])) OR “standard error of measurement”[tiab] OR sensitiv*[tiab] OR responsive*[tiab] OR ((minimal[tiab] OR minimally[tiab] OR clinical[tiab] OR clinically[tiab]) AND (important[tiab] OR significant[tiab] OR detectable[tiab]) AND (change[tiab] OR difference[tiab])) OR (small*[tiab] AND (real[tiab] OR detectable[tiab]) AND (change[tiab] OR difference[tiab])) OR meaningful change[tiab] OR “ceiling effect”[tiab] OR “floor effect”[tiab] OR “Item response model”[tiab] OR IRT[tiab] OR Rasch[tiab] OR “Differential item functioning”[tiab] OR DIF[tiab] OR “computer adaptive testing”[tiab] OR “item bank”[tiab] OR “cross-cultural equivalence”[tiab])

NOT

(“addresses”[Publication Type] OR “biography”[Publication Type] OR “case reports”[Publication Type] OR “comment”[Publication Type] OR “directory”[Publication Type] OR “editorial”[Publication Type] OR “festschrift”[Publication Type] OR “interview”[Publication Type] OR “lectures”[Publication Type] OR “legal cases”[Publication Type] OR “legislation”[Publication Type] OR “letter”[Publication Type] OR “news”[Publication Type] OR “newspaper article”[Publication Type] OR “patient education handout”[Publication Type] OR “popular works”[Publication Type] OR “congresses”[Publication Type] OR “consensus development conference”[Publication Type] OR “consensus development conference, nih”[Publication Type] OR “practice guideline”[Publication Type]) NOT (“animals”[MeSH Terms] NOT “humans”[MeSH Terms])

**Search strategy Embase**

(Continuity of care or Continuity of health care or Continuity of healthcare or Continuity of patient care or Continuity of service or Continuity of services or Care continuity or Healthcare continuity or Service continuity or Services continuity or Coordination of care or Coordination of health care or Coordination of healthcare or Coordination of patient care or Coordination of service or Coordination of services or Care coordination or Healthcare coordination or Service coordination or Services coordination or Co-ordination of care or Co-ordination of health care or Co-ordination of healthcare or Co-ordination of patient care or Co-ordination of service or Co-ordination of services or Care co-ordination or Healthcare co-ordination or Service co-ordination or Services co-ordination or Coordinated care or Coordinated health care or Coordinated healthcare or Coordinated patient care or Coordinated service or Coordinated services or Co-ordinated care or Co-ordinated health care or Co-ordinated healthcare or Co-ordinated patient care or Co-ordinated service or Co-ordinated services or Coordinating care or Coordinating health care or Coordinating healthcare or Coordinating patient care or Coordinating service or Coordinating services or Co-ordinating care or Co-ordinating health care or Co-ordinating healthcare or Co-ordinating patient care or Co-ordinating service or Co-ordinating services or Integration of care or Integration of health care or Integration of healthcare or Integration of patient care or Integration of service or Integration of services or Care integration or Healthcare integration or Service integration or Services integration or Integrated care or Integrated health care or Integrated healthcare or Integrated patient care or Integrated service or Integrated services or Integrating care or Integrating health care or Integrating healthcare or Integrating patient care or Integrating service or Integrating services or Patient centered care or Patient centered health care or Patient centered healthcare or Patient centered service or Patient centered services or Patient centred care or Patient centred health care or Patient centred healthcare or Patient centred service or Patient centred services or Patient focused care or Patient focused health care or Patient focused healthcare or Patient focused service or Patient focused services or Case management).ti,ab.

AND

exp questionnaire/ OR exp "named inventories, questionnaires and rating scales"/ OR exp psychometry/ OR exp outcome assessment/ OR exp validity/ OR exp reliability/ OR ((questionnaire or named inventory or reliability or rating scale or psychometry or outcome assessment or validity).tw.)

**Search strategy PsycInfo**

(Continuity of care or Continuity of health care or Continuity of healthcare or Continuity of patient care or Continuity of service or Continuity of services or Care continuity or Healthcare continuity or Service continuity or Services continuity or Coordination of care or Coordination of health care or Coordination of healthcare or Coordination of patient care or Coordination of service or Coordination of services or Care coordination or Healthcare coordination or Service coordination or Services coordination or Co-ordination of care or Co-ordination of health care or Co-ordination of healthcare or Co-ordination of patient care or Co-ordination of service or Co-ordination of services or Care co-ordination or Healthcare co-ordination or Service co-ordination or Services co-ordination or Coordinated care or Coordinated health care or Coordinated healthcare or Coordinated patient care or Coordinated service or Coordinated services or Co-ordinated care or Co-ordinated health care or Co-ordinated healthcare or Co-ordinated patient care or Co-ordinated service or Co-ordinated services or Coordinating care or Coordinating health care or Coordinating healthcare or Coordinating patient care or Coordinating service or Coordinating services or Co-ordinating care or Co-ordinating health care or Co-ordinating healthcare or Co-ordinating patient care or Co-ordinating service or Co-ordinating services or Integration of care or Integration of health care or Integration of healthcare or Integration of patient care or Integration of service or Integration of services or Care integration or Healthcare integration or Service integration or Services integration or Integrated care or Integrated health care or Integrated healthcare or Integrated patient care or Integrated service or Integrated services or Integrating care or Integrating health care or Integrating healthcare or Integrating patient care or Integrating service or Integrating services or Patient centered care or Patient centered health care or Patient centered healthcare or Patient centered service or Patient centered services or Patient centred care or Patient centred health care or Patient centred healthcare or Patient centred service or Patient centred services or Patient focused care or Patient focused health care or Patient focused healthcare or Patient focused service or Patient focused services or Case management).ti,ab.

AND

exp measurement/ OR exp test construction/ OR exp interrater reliability/ OR exp statistical analysis/
